# Progressive Failure Simulation of Notched Tensile Specimen for Triaxially-Braided Composites

**DOI:** 10.3390/ma12050833

**Published:** 2019-03-12

**Authors:** Zhenqiang Zhao, Haoyuan Dang, Jun Xing, Xi Li, Chao Zhang, Wieslaw K. Binienda, Yulong Li

**Affiliations:** 1 Department of Aeronautical Structure Engineering, Northwestern Polytechnical University, Youyi West Road 127#, Xi’an 710072, Shaanxi, China; zhaozhenqiang@mail.nwpu.edu.cn (Z.Z.); haoyuandang@mail.nwpu.edu.cn (H.D.); xingjun_acc@caac.gov.cn (J.X.); xili@mail.nwpu.edu.cn (X.L.); liyulong@nwpu.edu.cn (Y.L.); 2 Shaanxi Key Laboratory of Impact Dynamics and Its Engineering Applications, Youyi West Road 127#, Xi’an 710072, Shaanxi, China; 3 Joint International Research Laboratory of Impact Dynamics and Its Engineering Applications, Youyi West Road 127#, Xi’an 710072, Shaanxi, China; 4 Airworthiness Certification Center, Civil Aviation Administration of China, Beijing 100102, China; 5 Faculty of Aerospace Engineering, Delft University of Technology, 2629 Delft, The Netherlands; 6 Department of Civil Engineering, The University of Akron, Akron, OH 44325, USA; wbinienda@uakron.edu

**Keywords:** braided composites, mesoscale model, notched specimen, damage evolution

## Abstract

The mechanical characterization of textile composites is a challenging task, due to their nonuniform deformation and complicated failure phenomena. This article introduces a three-dimensional mesoscale finite element model to investigate the progressive damage behavior of a notched single-layer triaxially-braided composite subjected to axial tension. The damage initiation and propagation in fiber bundles are simulated using three-dimensional failure criteria and damage evolution law. A traction–separation law has been applied to predict the interfacial damage of fiber bundles. The proposed model is correlated and validated by the experimentally measured full field strain distributions and effective strength of the notched specimen. The progressive damage behavior of the fiber bundles is studied by examining the damage and stress contours at different loading stages. Parametric numerical studies are conducted to explore the role of modeling parameters and geometric characteristics on the internal damage behavior and global measured properties of the notched specimen. Moreover, the correlations of damage behavior, global stress–strain response, and the efficiency of the notched specimen are discussed in detail. The results of this paper deliver a throughout understanding of the damage behavior of braided composites and can help the specimen design of textile composites.

## 1. Introduction

Carbon fiber reinforced composite materials has some distinctive features in physical, mechanical, and thermal properties, such as high stiffness and strength to weight ratio, excellent resistance to fatigue, and corrosion. Traditional carbon fiber composite structures have layers of unidirectional fiber lamina and each layer can have a different direction of fiber lay-up, which is able to produce desired specific mechanical properties. However, a weak interlaminar plane where damage can initiate and cause delamination as in case of foreign object impact has limited the use of fiber composites in a variety of structures [1,2].

**Figure 1:**
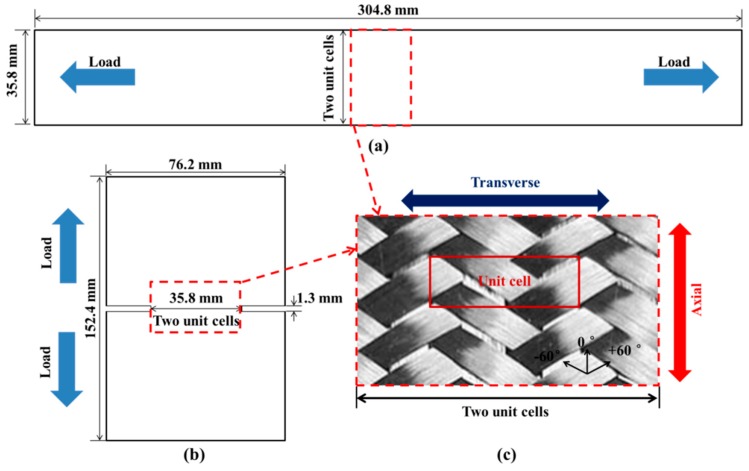
(**a**) Dimensions of the straight-side coupon. (**b**) Dimensions of double edge notch specimen. (**c**) Representative architecture of triaxially-braided composite.

Textile composites such as braided or woven composites are known to have excellent damage tolerance and impact resistance and are increasingly used in aircraft structures [3]. For example, the two-dimensional triaxially-braided composite is introduced to fabricate the engine fan case structure, which is mainly designed to contain the fan blade and its fragments during a blade failure event. Apart from its superior impact resistance property [4], the two-dimensional triaxially-braided composite also shows excellent specific energy absorption property and is considered as an alternative material system for front rail structures of vehicles [5,6]. Two-dimensional triaxially-braided fabrics are made by three distinct sets of yarns, which are intertwined to form a single layer of fabrics. Figure 1c shows the architecture of a typical 0°/± 60° braided fabric, bias fiber bundles undulate over and under each alternatively, while 0° yarns are straight and define the axial direction of the composite. The rectangle in Figure 1c indicates the size of a unit cell, which is considered as the smallest repeating element of a composite that can represent the composite’s geometric features in particular and its mechanical response as a whole. The length of a unit cell is the axial distance between center lines of two neighboring bias yarns, and the width is twice the transverse distance between the center lines of two neighboring axial yarns.

Due to the more complicated mesoscopic structure, the complexity of deformation and damage process for textile composites is greatly increased compared to that of laminates. Thus, the determination of mechanical properties for textile composites has drawn a lot of attention and raised significant challenges on the experiment techniques [7,8,9]. This paper focuses mainly on the tension failure behavior of triaxially-braided composite and investigates specifically the progressive failure process of a notched tensile specimen.

Waas and coworkers [7,10,11] studied extensively the compressive properties of a 0°/± 45° triaxially-braided composite using experimental, analytical, and numerical approaches. Goldberg et al. [12] identified that a 0°/± 60° braided composite offers improved impact resistance because of its quasi-isotropic nature (properties are balanced in all directions). Littell [13] and Kohlman et al. [14] studied experimentally the mechanical performance of a 0°/± 60° triaxially-braided composite using different kinds of experimental methods. Littell [13] conducted comprehensive tests to measure the quasi-static responses of triaxially-braided composites, including tension, compression, and shear. Littell’s results led to the conclusion that there were different damage mechanisms affecting the material response, including inherent damage accumulations (fiber bundle cracking and interface delamination) and geometry-induced premature failure behaviors (free-edge effect induced edge delamination). The presence of premature edge damage behavior in the standard straight-sided coupon specimen results in lower measured mechanical properties of the material.

For composite materials, it is difficult to avoid the possible premature failure caused by interlaminar stress concentration at the free edges of the specimen, which is more thought-provoking to accurately test the textile or braided composites. One major limitation is the local variation of properties for the fabrics since the methods for calculating lamina properties rely on the assumption of homogeneous strain and stress distribution in a uniaxial specimen [14]. For the triaxially-braided composite, the internal damage and its propagation depend significantly on the mesoscopic architecture of the material; the initiation of new damage will cause redistribution of internal loads, resulting in an inhomogeneous stress state. Through a combined experimental and numerical approach, Zhang et al. [15] investigated the mechanism of free-edge effect and the size-dependent mechanical properties of triaxially-braided composites. It was identified that the free-edge effect is an elastic behavior resulting from the termination of bias fiber bundles and affecting continuously the material response. Kueh et al. [16] identified the relationship of effective elastic properties of triaxially-braided composite against specimen size using an analytical approach.

To examine the realistic effective strength properties of the triaxially-braided composite, Kohlman et al. [14] designed several kinds of improved specimens to measure the mechanical properties of 0°/± 60° triaxially-braided composite, including both tube and notch geometries. The results further prove the sensitivity of measured properties to specimen shape and the significance of free-edge effect in triaxially-braided composites. It was also concluded that the notched coupon specimen produces higher measured strength values because of the enforced tensile failure of fiber bundles at the notched gauge section. Compared with the straight-sided coupon specimen, the damage behavior of notched specimens is more complicated, due to the presence of stress concentration in the notched zone. Thus, it is necessary to develop representative numerical models to analyze and elucidate the progressive failure behavior of notched tensile specimen. Using a numerical model as a virtual testing tool of composites can also provide insights in revealing the localized mechanical response and exploring damage mechanism at meso and microscopic scale, which can then facilitate the development of experimental techniques.

Mesoscale finite element (FE) is known for its capability in predicting the local response and damage events of textile composite [17,18,19]. Lomov et al. [20] conducted a comprehensive study on the mesoscale finite element modeling approach of textile composites. Especially for triaxially-braided composites, Zhang et al. [15,18] established a mesoscale finite element framework, with emphasize on imposing representative loading/boundary conditions against an experimental set-up; Zhao [21] utilizes the mesoscale FE model to study intensively the failure behavior under transverse tension and compression, and its damage behavior under high-speed impact has been exactly captured by proposing a multiscale modeling framework based on a fully validated mesoscale FE model [22]. Apart from these, the fracture process of triaxially-braided composite for straight-sided coupon specimens also can be simulated by means of the mesoscale FE model [23,24].

However, there is no reported work applying the mesoscale FE model to the analysis of specimens with more complicated shapes, e.g., notched specimen, tube specimen, and specimen with hole. This limits the confidence of the community on the feasibility of meso-FE model for virtual testing. On the other hand, the presence of challenges in characterizing the mechanical properties of 2DTBC requires further efforts in investigating the failure mechanism and optimizing the test specimens. Thus, in this work, the mesoscale finite element method with three-dimensional damage model is introduced to investigate the progressive failure behavior of notched specimen of the triaxially-braided composite under axial tension. The presented model intends to simulate the damage initiation, damage propagation, and ultimate fracture of the notched specimen, as well as to predict the effective strength of the triaxially-braided composite. The results demonstrate the accessibility of using mesoscale finite element model as virtual testing for textile composites, which can significantly enhance the design efficiency of composite structures. This research paper firstly describes the material system and experimental details followed by the progressive damage model of the composite (which consists of damage initiation criteria and its subsequent evolution). Then, the mesoscale finite element model is introduced. The Section 5 of this paper examines the capability of the mesoscale model through correlation with experiments conducted by Kohlman et al. [14] and presents the predicted results of local initiation and progression of damage. Additionally, the parameters study and geometric characteristic analysis of notched specimen are also discussed in this section. The conclusions are listed in the last section of this paper.

## 2. Materials and Experiment

The 0°/± 60° triaxially-braided composite studied in this paper was fabricated with Toray 24 K T700 s axial tows and Toray 12 K T700 s bias tows. Epon’s 862 epoxy resin was chosen as matrix material, which is a thermoset resin with low viscosity. The composite panels were processed through resin transfer molding (RTM). Table 1 presents the properties of each component, which are obtained from Littell [13].

The sample considered in the present study is a single-layer panel (a composite containing only one braided ply through thickness) with double edge notches (shown in Figure 1b), which was designed and tested by Kohlman [14] to address deficiencies of straight-side tension coupons. The thickness of the single-layer specimen is 0.65 mm. Other dimensions of the notched and straight-sided coupon specimens are shown in Figure 1. A diamond saw was used to cut the notches and kept the same width of gauge region to compare with the test results of straight-sided coupon specimens. Tensile tests were performed using a servohydraulic tension/torsion test frame capable of loading to 220KN (MTS Systems Corporation, Eden Prairie, MN, USA). The specimens were stretched under displacement-controlled load until fracture of the specimen. 3D digital image correlation (GOM, Braunschweig, Germany) technique was used to obtain the full field displacement and strain data on the surface of the notched specimens.

## 3. Progressive Damage Model of Braided Composite

The fiber bundle of textile composites is generally considered as a transversely isotropic unidirectional lamina in numerical simulation [25,26,27]. In this part, a progressive damage model of the unidirectional lamina is formulated in terms of damage initiation and damage evolution. This research aims to investigate the internal damage initiation and propagation of a notched specimen, therefore material failure (element deletion) is not introduced in this damage model.

### 3.1. Damage Initiation

A three-dimensional failure criterion for the fiber bundle was adopted based on Hashin’s [28] and Hou’s [29] criteria and was incorporated with continuum damage laws. Four distinct failure modes are considered: fiber tensile failure, fiber compression failure, matrix tension failure, and matrix compression failure. The damage initiation criteria are formulated below.

For fiber tension failure (*σ*_11_ > 0),

(1) $$f_{1t}={\left(\frac{\sigma_{11}}{S_{1t}}\right)}^{2}+\alpha \left(\frac{\sigma_{12}+\sigma_{31}}{S_{12}}\right)\geq 1$$

For fiber compression failure (*σ*_11_ < 0),

(2) $$f_{1c}={\left(\frac{\sigma_{11}}{S_{1c}}\right)}^{2}\geq 1$$

For matrix tension failure (*σ*_22_ > 0),

(3) $$f_{2t}={\left(\frac{\sigma_{22}}{S_{2t}}\right)}^{2}+{\left(\frac{\sigma_{12}}{S_{12}}\right)}^{2}+{\left(\frac{\sigma_{23}}{S_{23}}\right)}^{2}\geq 1$$

For matrix compression failure (*σ*_22_ < 0),

(4) $$f_{2c}=\frac{1}{4}{\left(\frac{-\sigma_{22}}{S_{12}}\right)}^{2}+\frac{\sigma_{22}}{S_{2c}}\left({\left(\frac{S_{2c}}{2S_{12}}\right)}^{2}-1\right)+{\left(\frac{\sigma_{12}}{S_{23}}\right)}^{2}\geq 1$$

where *f*_1*t*_, *f*_1*c*_, *f*_2*t*_, and *f*_2*c*_ are failure indices corresponding to each damage mode, respectively. The first subscripts, 1, 2, and 3, indicate the fiber axial direction, in-plane transverse direction, and out-of-plane direction, respectively. When the stress state of an element make one of the four failure indices larger than 1, the corresponding damage mode will be initiated in this element and constitutive law entering the stage of damage evolution. *S*_1*t*_, *S*_1*c*_, *S*_2*t*_, *S*_2*c*_, *S*_12_, and *S*_23_ are axial tensile strength, axial compressive strength, transverse tensile strength, transverse compressive strength, longitudinal shear strength, and transverse shear strength of the fiber bundle. *α* is the shear failure coefficient which plays an important role in failure prediction of textile composite. The previous research conducted by Zhang et al. [18] indicated that the coefficient *α* has an obvious impact on the global stress–strain response and mainly on the failure prediction. The value of *α* was determined to be 0.06 for fiber bundles of triaxially-braided composite through correlation with an experimental ultimate strength of straight-sided coupon specimens [18,22]. The same value of parameter *α* is adopted in the present study in consideration that the studied materials are totally the same.

### 3.2. Damage Evolution

For the damage evolution behavior, the Murakami–Ohno [30] damage theory is adopted to predict the post-peak softening, and the crack band model developed by Bazant and Oh [30] is also employed to mitigate the mesh size dependency of the proposed mesoscale model in this study. A characteristic element length is introduced into damage evolution expression, aiming to dissipate the constant energy release rate per unit area in the solid element [31,32], and the element dissipated energy can be expressed as

(5) $$G_{f,I}=\frac{1}{2}\sigma_{eq}^{f}\varepsilon_{eq}^{f}l_{c}$$

where *l_c_* is the characteristic length of element, which calculates by extracting the cubic root of the volume of each element; *G_f_*_,*I*_ is fracture energy of fiber bundles corresponding to the specific damage mode *I*. The values for the fracture energies of axial and bias fiber bundles used in this study (listed in Table 2) were cited from Li et al. [17]. $\sigma_{eq}^{f}$ and $\varepsilon_{eq}^{f}$ are the equivalent peak stress and equivalent failure strain, respectively. The evolution of each damage variable is governed by an equivalent displacement expressed by the following equation.

(6) $$d_{I}=\frac{\delta_{I,eq}^{f}\left(\delta_{I,eq}-\delta_{I,eq}^{0}\right)}{\delta_{I,eq}\left(\delta_{I,eq}^{f}-\delta_{I,eq}^{0}\right)},\;I=ft,\;fc,\;mt,\;mc$$

where $\delta_{I,eq}^{f}$ is the fully damaged equivalent displacement of the corresponding failure mode and $\delta_{I,eq}^{0}$ is the equivalent displacement at which the failure criterion is satisfied. For a certain failure mode, the equivalent displacement used in the initiation criteria is expressed in terms of the components corresponding to the effective stress components. Detailed algorithm equations of equivalent displacement and stress for each failure mode can be found in Zhang and coworkers’ work [18]. As material parameters of fiber bundles, $\delta_{I,eq}^{0}$ and $\delta_{I,eq}^{f}$ be computed by the following equations.

(7) $$\delta_{I,eq}^{f}=\frac{2G_{f}}{\sigma_{I,eq}^{0}}$$

(8) $$\delta_{I,eq}^{0}=\frac{\delta_{I,eq}}{\sqrt{f_{I}}}$$

Here, $\sigma_{I,eq}^{0}$ denotes the equivalence stress when each kind of damage criteria is satisfied. Meanwhile, the value of *f_I_* can be obtained from Equations (1)–(4).

To simulate the softening process of damage element, a second-order symmetric tensor is used to describe the damage state. The corresponding damaged compliance matrix *S*(*d*) is obtained as Equation (9), and the damaged stiffness matrix *C*(*d*) is the inverse of *S*(*d*).

(9) $$S\left(d\right)=\left[\begin{array}{cccccc} \frac{1}{d_{f}E_{11}} & -\frac{\nu_{21}}{E_{22}} & -\frac{\nu_{31}}{E_{33}} & & & zero\\ -\frac{\nu_{12}}{E_{11}} & \frac{1}{d_{m}E_{22}} & -\frac{\nu_{32}}{E_{33}} & & & \\ -\frac{\nu_{13}}{E_{11}} & -\frac{\nu_{23}}{E_{22}} & \frac{1}{E_{33}} & & & \\ & & & \frac{1}{d_{f}d_{m}G_{12}} & & \\ & & & & \frac{1}{d_{m}G_{23}} & \\ zero & & & & & \frac{1}{d_{f}G_{13}} \end{array}\right]$$

where *d_f_* and *d_m_* are global damage variables associated with fiber and matrix failure, which are introduced to control the degree of stiffness degeneration of damaged elements, and also satisfy the following equations, respectively.

(10) $$d_{f}=1-\min\left\{\max\left(d_{ft},d_{fc}\right),\gamma_{f}\right\}$$

(11) $$d_{m}=1-\min\left\{\max\left(d_{mt},d_{mc}\right),\gamma_{m}\right\}$$

Here *γ_f_* and *γ_m_* are defined as the damage thresholds of global fiber and matrix damage, respectively. Numerically, the nonzero constants *γ_f_* and *γ_m_* address the singularity issue, and physically represent the effective resultant resistance of the homogeneous damaged elements. The effect of *γ_f_* and *γ_m_* on the global stress–strain responses will be discussed in a later section. Also, the Duvaut and Lions regularization model [33] is applied to promote the numerical computation and smooth the stiffness degradation process.

### 3.3. Cohesive Element Model for Interface

Interface is the bridge between the fiber bundle and matrix, which determines how stresses are transferred. The damage status of interface influences significantly the damage initiation and propagation of the composite material [34]. Littell [13] indicates that failure in the transverse tests was a result of edge delamination which occurred quickly and propagated along the bias fibers; and in the axial tensile direction, the subsurface delamination caused the nonlinearities in the global stress–strain response curves. In this study, the tow-to-tow interface is simulated by using the cohesive zone modeling approach, which has been embedded into ABAQUS as an optional element type. The responses of cohesive elements are governed by a typical bilinear traction–separation law, and a quadratic nominal stress criterion is used to describe interfacial damage initiation [32,34,35]. Besides, a power law criterion is adopted, which claims that failure under mixed-mode conditions is governed by a second-order power law interacting of the energies required to cause failure in the individual (normal and two shear) modes. The quadratic nominal stress criterion for damage initiation and a power law criterion for failure are represented in Equations (12) and (13).

(12) $${\left(\frac{\langle t_{n}\rangle }{t_{n}^{0}}\right)}^{2}+{\left(\frac{\langle t_{s}\rangle }{t_{s}^{0}}\right)}^{2}+{\left(\frac{\langle t_{t}\rangle }{t_{t}^{0}}\right)}^{2}=1$$

(13) $${\left(\frac{G_{n}}{G_{n}^{c}}\right)}^{2}+{\left(\frac{G_{s}}{G_{s}^{c}}\right)}^{2}+{\left(\frac{G_{t}}{G_{t}^{c}}\right)}^{2}=1$$

where *t_n_* denotes the traction normal stress, and *t_s_* and *t_t_* denote shear stresses. The Macaulay brackets are used to signify that a pure compressive deformation or stress state does not initiate damage. $t_{n}^{0}$, $t_{s}^{0}$, and $t_{t}^{0}$ represent the interface strength in normal and two shear directions. Similarly, *G_n_*, *G_s_*, and *G_t_* refer to the work done by the traction and its conjugate relative displacement in the normal, first, and second shear directions, respectively; and $G_{n}^{c}$, $G_{s}^{c}$, and $G_{t}^{c}$ are critical fracture energies required to cause failure in each of the three directions. Table 3 presents the interface properties, and the values of interface strengths and fracture toughness, which are cited from Zhang et al. [18]. Detailed formulations of the mixed-mode cohesive zone model can be found in the ABAQUS user’s manual.

## 4. Finite Element Model Development

The mesoscale finite element (FE) model simulates explicitly the fiber bundles of a braided composite structure and defines locally the realistic local fiber volume ratios and bundle orientations of the impregnated bundles. The advantage of the mesoscale model is its ability to analyze the local damage and failure of each component implemented individually through specific failure models for the various constituents and to predict the response of each constituent and their contribution to the global behavior. In this work, a mesoscale finite element model is introduced to study the internal damage and failure mechanism of the notched tensile specimen for 0°/± 60° triaxially-braided composite.

**Figure 2:**
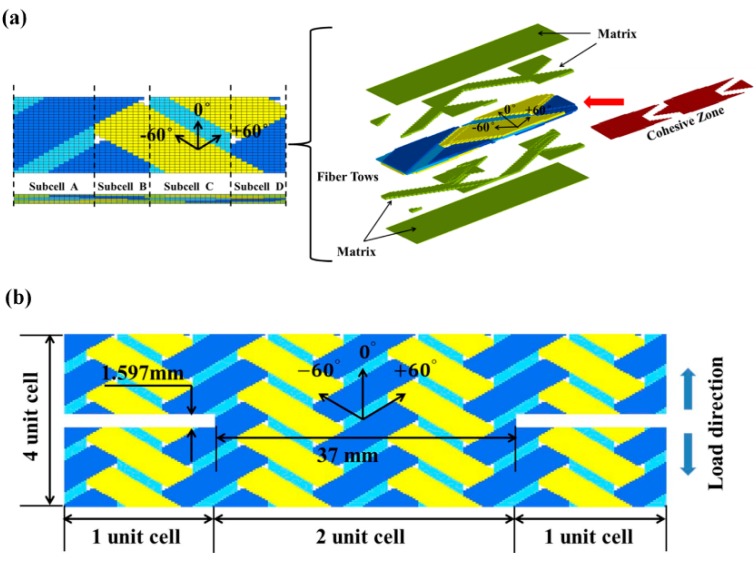
(**a**) Composition of a unit cell finite element model. (**b**) Finite element mesh of axial tension notched model.

Based on the geometric parameters identified by Zhang et al. [18], a FE model of a single unit cell, which can represent the composite’s geometric features, was generated using 8-node solid element. As shown in Figure 2a, the mesh of a unit cell was constructed through TexGEN software by keying the dimensions of the unit cell and fiber bundles. In Figure 2, the axial fiber bundle, +60° and −60 ° bias fiber bundles and matrix elements, which fill the space between fiber bundles to form plate, are represented by light blue, dark blue, yellow, and green colors, respectively. Cohesive element layers (colored red), which have the same in-plane size as brick elements but zero thickness, are manually inserted between each two fiber bundles, and fiber bundle and matrix, see Figure 2a. It should be pointed out that the pure matrix is modeled as an elastic perfectly-plastic material. It is assumed that the pure matrix elements will not fail before the fracture of the specimen, due to the much larger failure strain of matrix than that of the fiber bundle. The resultant mesh may have different fiber bundle volume values than the real material, so the fiber volume ratio in each fiber bundle is adjusted to match the real fiber volume content. As identified by Zhang et al. [18], the realistic fiber volume ratios for axial and bias fiber bundles are 77% and 74.5%, respectively. The resultant fiber volume ratio in the present FE model is 86% for the axial fiber bundles and 69% for the bias fiber bundles. Mechanical properties of fiber bundles are listed in Table 4 referring to Zhang’s work [18] for the same materials.

Figure 2b shows the detailed dimensions of the FE model for the notched specimen. The FE model of the notched tension specimen consists of 208,000 linear brick elements (C3D8R) and 29,412 eight-node three-dimensional cohesive elements (COH3D8), with four unit cells through the width and length direction, respectively. Two whole unit cells (37 mm) are assembled through the width direction of the notched gauge for the axial tension model, which is consistent with the experimental specimen. The length of the finite element model is 20.68 mm with four unit cells for axial tension, which are shorter than the gauge length of experimental specimens fabricated by Kohlman [14] (Figure 1b). These reasonable simplifications of model size are intended to reduce the computational time, in consideration of the minor effect of the remote sections. The numerical models are solved by ABAQUS Explicit using a 24-core workstation and each job costs ~20 h. By evaluating the various energies generated during the computation process, the accumulated kinetic energy was always less than 1% of the internal energy of the model, which ensures a quasi-static loading status of the problem.

## 5. Results and Discussion

In this section, the damage initiation and propagation behavior of the notched tension specimen modeled using the proposed mesoscale FE scheme is correlated with experimental results. The effect of specimen geometry on the internal damage evolution behavior and the effective strength of this notched specimen are further discussed through numerical parametric studies. These results show the applicability and reliability of the mesoscale FE model for failure study of braided composites.

### 5.1. Model Correlation

**Figure 3:**
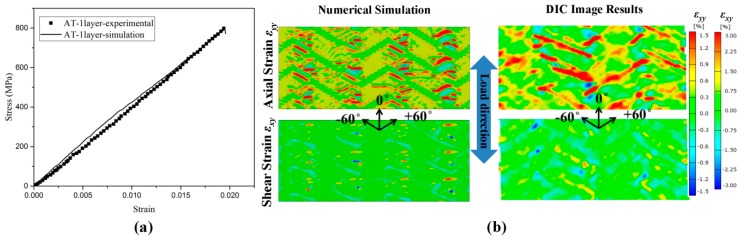
Comparison of the experimental and numerical predicted results for straight-side coupon specimen. (**a**) Global stress–strain curves. (**b**) Distribution of the axial and in-plane shear strain at global strain level of 2.0%.

In our previous works [15,18,21,22], the failure behavior of straight-side coupon specimens of 2DTBC has been intensively studied. Figure 3 shows the comparison of the experimental and numerical predicted results for the single-layer straight-side coupon specimen under axial tension. The results indicate that the mesoscale FE model can not only predict well the global stress–strain curve, but also the strain distributions which are highly sensitive to the local braided architecture.

The applicability of the mesoscale FE model is further demonstrated through the model validation for the notched specimen. For both the experimental characterization and numerical simulations, the effective strength of the tensile specimen is determined as the ratio of total reaction force at the loading section against the cross-section area at the gauge section (two unit cells wide for both notched and straight-sided coupon specimen). Table 5 compares the numerical predicted and experimental measured effective strength of triaxially-braided composite under axial tension, for both straight-sided coupon and notched specimens. The measured strength values of the straight-sided coupon and notched specimen are referred to as Kohlman [14]. It is evident from Table 5 that the mesoscale FE model predicted effective strength values are in good agreement with the experimental values, suggesting the accuracy of this modeling scheme. The effective strength of the notched specimen tends to be lower than the straight-sided coupon specimen, which is due to the presence of stress concentration at the notched section.

**Figure 4:**
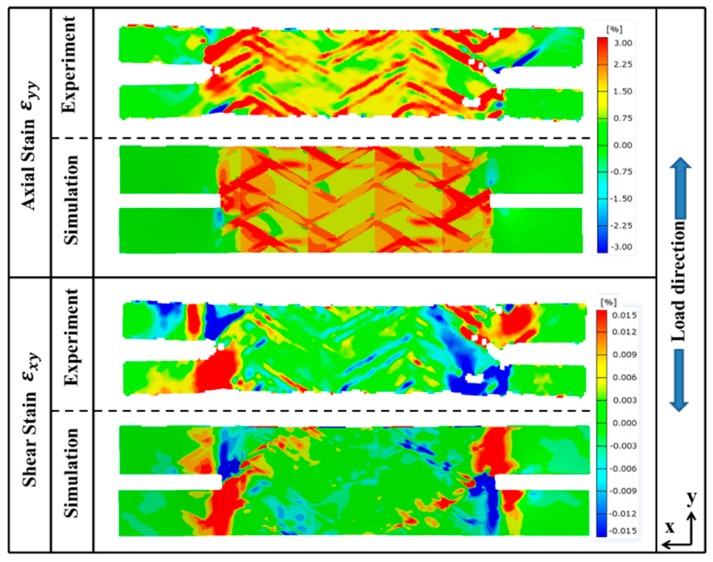
Comparison of numerical predicted and experimentally measured strain contours of the notched specimen under axial tension.

To further demonstrate the reliability of the meso-FE model, the numerically predicted strain distributions are compared with DIC results. As seen in Figure 4, the simulation results match well with the local strain distribution of the notched specimen and additionally capturing the local strain concentration around the notch area and the diagonal distribution along the bias fiber bundles. The axial strain (*ε_yy_*) and shear strain (*ε_xy_*) distributions show higher strain at the pure matrix zone between bias fiber bundles, which coincides with the damage propagation paths of fiber bundle damage. The damage of fiber bundles will induce the decrease of fiber’s capability in carrying the load and as a result, more load will be transferred to the matrix material, thereby causing the strain concentration in this area (red and blue in Figure 4). On the other hand, the shear strain shows more obvious strain concentration effect at the notched section. Unlike the traditional isotropic material where strain concentration effect propagates in a circular shape, the shear strain concentration effect of this braided composite propagates along the fiber bundle directions. The mesoscale FE model simulations capture the strain distribution and its magnitude accurately for the axial tension of the notched specimen. The result also reflects the advantages of mesoscale finite element model in studying the internal damage of textile composite that is difficult to achieve from experiments.

Furthermore, another reason for the localized strain concentration effect is because of the lower local fiber volume ratio at the corresponding regions. The inconspicuous discrepancy between numerically predicted and experimental axial strain contours is very likely results from the idealization of the geometry in the FE model. However, the manufacturing defects like axial fiber bundle undulation are inevitable in actual specimens. The cutting process may also introduce unexpected fiber damage, which could lead to more significant strain concentration area near the notch. Regardless of these factors, the capability and accuracy of this 3D mesoscale model are highly acceptable.

**Figure 5:**
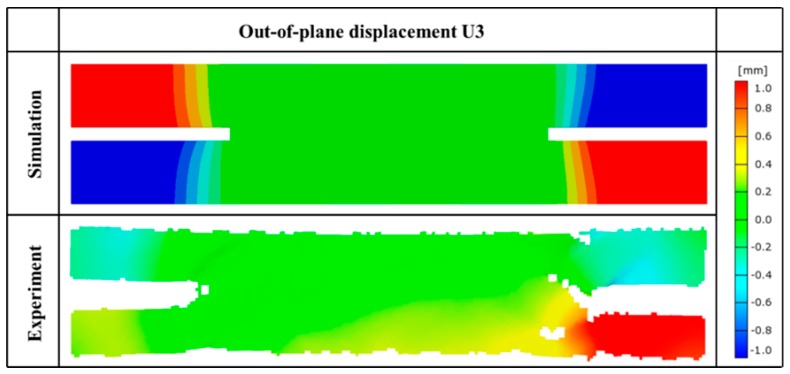
Comparison of numerical predicted and experimental measured out-of-plane displacement contours of notched tension specimen.

As mentioned before in the introduction section, both Littell [13] and Zhang et al. [15] observed out-of-plane warping behavior along the free edges in their tensile tests using the straight-side coupon specimen, which would lead to the premature damage initiation from the free edges. Kolhman et al. [14] also discovered the same phenomena in the notched specimen. Figure 5 compares the out-of-plane displacement (U3) contours from simulation and experiment for the notched tension test. The out-of-plane displacement is formed due to the tension–torsion coupling resulting from the termination of fiber bundles at the free edges. In the simulation results, the specimen shows the antisymmetric distribution of out-of-plane warping at the four corners of the notched specimen, which is related to the antisymmetric mesoscopic structure of this material. In addition, no obvious out-of-plane deformation was observed in the gauge section of the specimen indicates that the failure of the specimen is not affected by the free-edge effect. However, the out-of-plane deformation still consumes partially the energy from external loading, leading to a relatively lower measured modulus of the specimen. Thus, the notched specimen may not be suitable for modulus measurement. This is one of the contents which need to be studied in the future.

### 5.2. Progressive Damage Analysis

**Figure 6:**
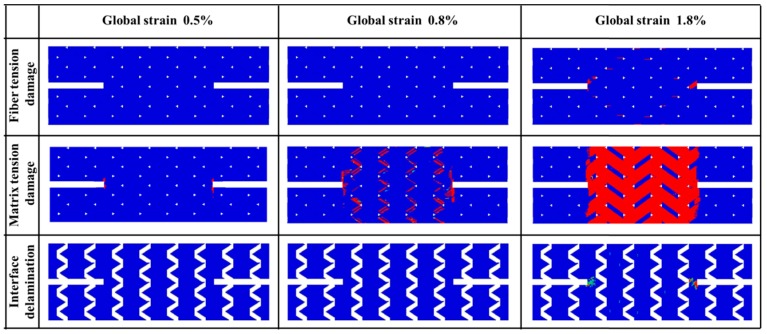
Comparison of numerical predicted damage development of axial tension.

The progressive damage process is then studied using the correlated mesoscale FE model, as shown in Figure 6. The status of damage corresponds to the value of the particular damage variable ranging from 0 to 1, where a value of 0 indicates that damage has not occurred yet while a value of 1 indicates that the element is totally damaged.

The matrix damage initiated at the global strain level of ~0.5% and spread to the whole central region at the global strain level of 0.80%. The distribution of internal damage inside the bundles reveals that there is no fiber tension damage occurring at these strain levels while matrix tension damage (matrix cracking in fiber bundles) is observed to start from the notched area. The matrix tension damage propagates along the notches and the bias fiber bundles; while in the meantime, in the central region matrix tension damage occurs mainly where bias fiber bundles intersect. The interfaces are found to be intact at the first two status of Figure 6 as there is no damage in cohesive elements. This behavior is similar to the observations during the test of straight-sided coupon specimen [13].

At the global strain level of 1.8%, unloading is identified followed by fiber tensile damage of axial fiber bundles and almost all elements of bias fiber bundles are dominated by matrix tension damage. The fiber tension damage initiates at the notched area same as the matrix tension damage. It is also found that interface failure appears only near the notches due to localized shear stress concentration. The inert of interface for all other areas indicates the excellent interface properties of this triaxially-braided composite, which is consistent with the previous experimental examinations where delamination is rarely observed during axial tension tests of this material [14].

**Figure 7:**
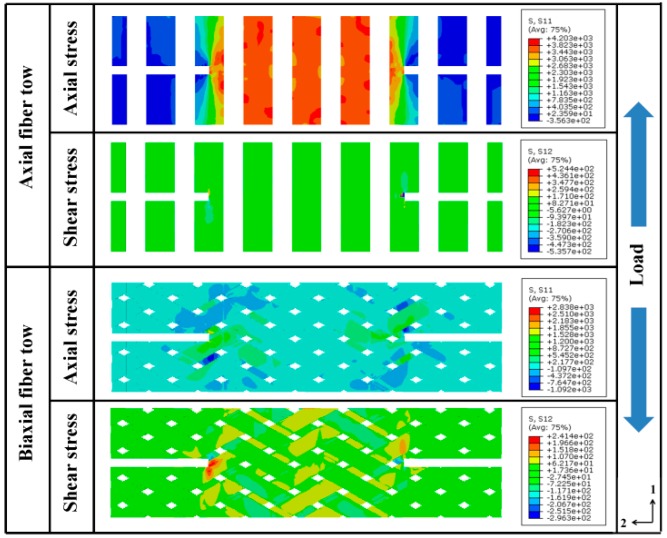
Numerical predicted stress contours before the instant of failure under axial tension.

Figure 7 shows the numerically predicted stress distributions of axial and biaxial tows before the specimen failure. The stresses on both sides of the notches are not symmetrical because of the asymmetry geometry feature. As seen from the stress contours, most of the applied loads are afforded by the axial bundles across the notched sections. Similar to the strain distribution contours in Figure 4, the shear stress concentration at the notched area is more significant than the normal stress. The shear stress concentration will then result in shear tension-dominated failure of axial fiber bundles at the notched area and tension dominated failure of axial fiber bundles at the central area, corresponding to the inclined (along bias fiber bundle direction) failure pattern of the two axial fiber bundles near the notches and horizontal failure pattern of fiber bundles at other region (fiber tension damage at global strain level 1.8% as shown in Figure 6). Similarly, the stress distribution of bias fiber bundles also explains the initiation and propagation behavior of matrix tension damage behavior at global strain levels 0.5% and 0.8% (Figure 6).

Overall, matrix tension damage appears firstly around the notched areas and propagates along the bias fiber bundles, resulting in a slight decrease of effective stiffness. With the increase of external loads, the fiber tension damage and delamination will initiate around the notches due to shear stress and strain concentration in this region. The notch-induced stress/strain concentration effect disturbs only a limited region of the specimen and the failure of the specimen is mainly due to normal tension stress-induced fiber tension damage in the axial fiber bundles. Thus, the measured tensile strength of a notched specimen can be representative and can be a lower bound of the realistic strength.

The damage areas at strain level 1.8% of Figure 6 are in good correlation with the strain concentration region of the experimental results reported by Kolhman et al. [14]. As observed by Kolhman, the highest strain appears near the notch tips, which mainly attribute to the fiber tension failure. The relatively high strain concentrated along bias fiber is a result of matrix damage within the fiber bundles. The results of this section demonstrate the capability of a mesoscale model in predicting the internal damage initiation and propagation behavior of textile composites using various specimen shapes.

### 5.3. Numerical Parametric Study

Numerical studies were carried out to further investigate the features of the proposed mesoscale FE model and how the specific material parameters contribute to the global response of the model. This is a difficult task mainly because of the tedious modeling process and the considerable computational quantity. Due to nonuniformity of the strain distribution for the entire specimen, smooth stress–strain curves can hardly be produced in the notched tension tests, which were used mainly for strength measurement in Kohlman’s work [14]. For numerical comparison, in the meso-FE simulations, stress–strain curves are generated based on the method of “digital strain gauge” proposed by Littell [13]. The macroscopic effective stress for the analyzed section is computed by determining the summation of the reaction forces on the face of the loaded cross-section divided by the area of the cross-section between two notches. The effective strain is calculated by dividing the relative displacement along the loading direction between two nodes by the initial distance between these two nodes before loading, where the relative displacement is calculated as the midpoints on each end (through load direction) section of the gauge region.

**Figure 8:**
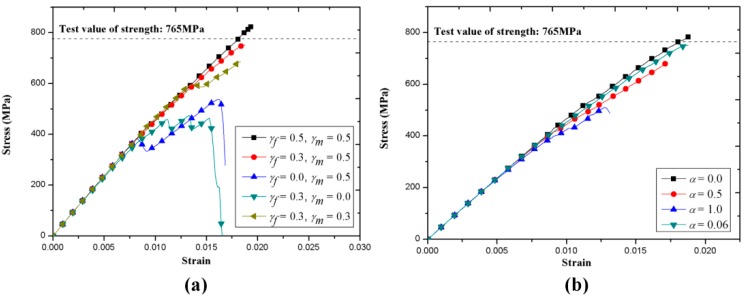
Numerical predicted stress-stain responses of notched tension specimen with: (**a**) different damage thresholds and (**b**) different shear failure coefficients.

Figure 8a shows the variation of global stress–strain responses with different damage thresholds (*γ_f_* and *γ_m_*). The dashed line in the figure corresponds to the experimental measured effective strength of this notched specimen by Kohlman [14]. As mentioned previously, *γ_f_* and *γ_m_* are the damage thresholds of *d_f_* and *d_m_*, which are the global damage variables associated with fiber-dominated and matrix-dominated failure, respectively. *d_f_* and *d_m_* control the descent of element stiffness along with damage accumulation, while *γ_f_* and *γ_m_* determine the extent of stiffness degradation. As we can see in Figure 8a, there is a sudden force drop with damage accumulation when *γ_f_* is lower than 0.3 and *γ_m_* is lower than 0.5, which is not physically consistent with the experimental stress–strain response of this material where the slope of the stress–strain curve tends to degrade gradually. Numerically, the stress–strain curves become smooth when *γ_f_* and *γ_m_* are larger than 0.3 and 0.5, respectively. Combined with the progressive damage behavior of this specimen discussed in the previous sections, the effect of *γ_f_* and *γ_m_* is concluded as follows; a smaller value of *γ_f_* leads to premature unloading of the stress–strain curve and *γ_m_* impacts the extent of nonlinearity of the curve. This is because the external load is mainly carried by axial fiber bundles under axial tension and the fiber tension damage mainly presences in the axial bundles. Then a rapid degradation of stiffness caused by fiber damage will result in a loss of load-bearing capacity instantaneously in the local area and correspond to a sharp drop of the stress–strain curve. On the other hand, *γ_m_* may not affect much the global responses at the initial stage due to the anisotropic feature of fiber bundles. However, as the matrix damage accumulates and propagates, *γ_m_* becomes more significant and could result in unexpected slope change followed by the initiation of fiber damage (see Figure 8a *γ_f_* = 0.3 and *γ_m_* = 0.5). Also, the predicted effective strength is found to be sensitive to the parameters *γ_f_* and *γ_m_*. In this work, the numerical predicted effective strength correlates with experimental results when *γ_f_* = 0.3 and *γ_m_* = 0.5.

The shear failure coefficient *α* controls the contribution of shear stress on fiber tension damage. The results exhibited in Figure 8b are similar to the conclusion for the same material reported in Zhang [18]. It is found that the effect of *α* on effective strength is not apparent when the value of *α* is lower than 0.5. As expected, the notch induced shear stress/strain concentration produces slightly higher sensitivity of the global stress–strain responses to the shear coefficient. The results of parameter analysis also declare that the present constitutive model of fiber bundles needs further improvement to enhance accuracy and applicability.

### 5.4. Effect of Specimen Shape

The mesoscale FE model proposed in this study can also be used to assess the rationality of the designed specimen. The effect of geometrical characteristics of the notched specimen on the test results was not considered by Kohlmen [14], due to the enormous consumption of time and availability of specimens. In this work, the correlated mesoscale FE model is employed to address this concern.

**Figure 9:**
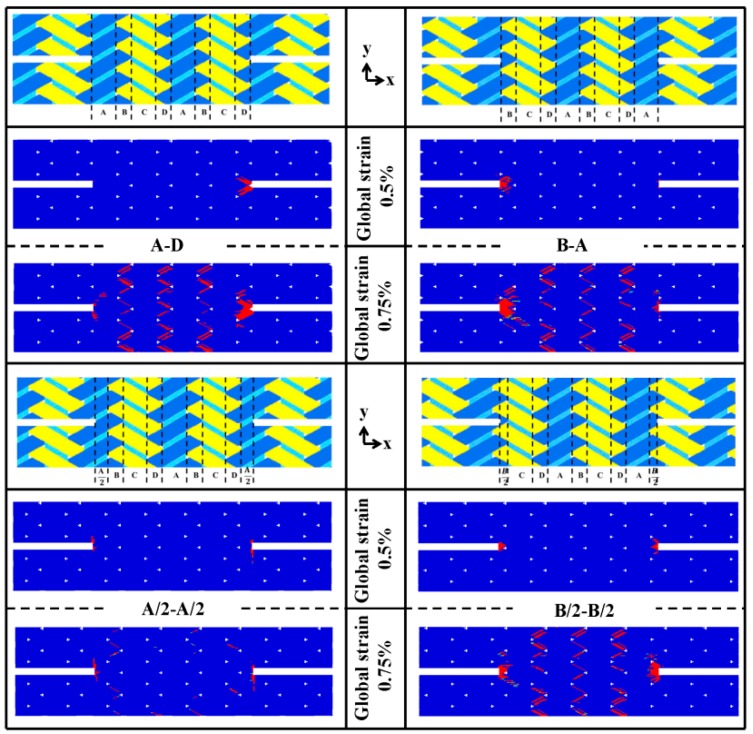
Numerical predictions of matrix damage in fiber bundles for notched tension specimen with different architectures across the gauge section.

Referring to the 0°/± 60° braided architecture shown in Figure 2a, a unit cell of this braided composite can be discretized into four adjacent subcell regions depending on the presence of axial and braided tows or lack thereof. A subcell-based modeling approach has been used by many researchers [12,36] to investigate the static and impact behavior of this triaxially-braided composite. Subcells A and C contain both axial and bias bundles while subcells B and D contain only bias bundles, so the distinction of fiber volume in each subcell leads to the different local mechanical properties in a unit cell. As from the top view and front-side view of the unit cell (Figure 2a), subcells C and D are antisymmetric against subcells A and B in the thickness direction. The section studies the relationship of different subcell distributions in the gauge region with damage evolution behavior and the ultimate strength property of the notched specimen. Figure 9 compares the geometry and damage contours of different notched specimens, where the subcells adjacent to the notches of the specimens are inequitable. The label above each specimen represents the two subcells which are closest to the two notches. For instance, “A-D” indicates that subcell A and D are located in the ends of gauge region while “A/2-A/2” expresses that half of subcell A connects with the notches.

As from Figure 9, the damages of all specimens initiate and concentrate at the notched area at a global strain level of 0.5%. For specimens “A-D” and “B-A”, the damage initiates and concentrates at one of the two notched zones, and the damage initiation locations of the two specimens are asymmetrically suggesting the asymmetric fabric architecture of the two specimens. This damage behavior is related to the fabrics subcells layout (Figure 9), where subcells A and C consist of both axial and bias bundles and are supposed to be stronger in the axial direction than subcells B and D. Thus, when subcells A/C and B/D are subjected to the same extent of load, subcells B and D are likely to damage earlier. Similarly, specimens “B/2-B/2” and “A/2-A/2” show symmetric damage progression behavior, as they both have the same fabric subcells layout at the notched area. Besides, comparing the specimens “B/2-B/2” and “A/2-A/2”, it is found that the presence of axial fiber bundles along the notches restrict the amount of matrix damage and its propagation in fiber bundles, especially obvious at the global strain level of 0.75% where the matrix damage area is much smaller than that in the other three specimens. However, matrix damage in fiber bundles has little influence on the axial stiffness of fiber bundles and shows the negligible impact to the ultimate strength of the notched specimen. Table 6 lists the predicted effective strength of the four different notched specimens shown in Figure 9. While all specimens have more or less the same effective strength, specimen “A/2-A/2” shows relatively lower strength attributing to the incomplete axial fiber bundles in the gauge region. In the numerical simulations, the crossing of the fiber damage through the width (*x*-direction) of a single fiber bundle corresponds to the fracture of the specimen. Thus, the presence of incomplete axial fiber bundles at both ends of the gauge region in specimen “A/2-A/2” results in an earlier failure of the specimen. It should be noted that the fiber volume ratio in the gauge region of these four kinds of specimens is the same to ensure the comparability.

**Figure 10:**
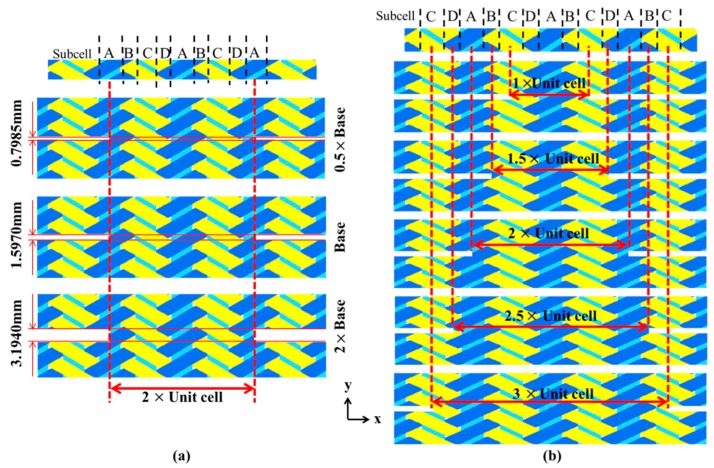
Mesoscale models of notched specimens with different geometrical characteristics. (**a**) Different notch sizes. (**b**) Different widths of the gauge section (with complete axial tows between notches).

To investigate the effect of notch geometry on the effective strength of the specimen numerically, specimens with three different notch sizes (dimension in *y*-direction as shown in Figure 10a) and five different widths of the gauge section (dimension in *x*-direction as shown in Figure 10b) were studied.

The numerically predicted effective strength results are also listed in Table 7, where the effective strength values are found to be insensitive to the height (*y*-direction value) of the notch size for the cases studied in this work. However, the axial ultimate strength for the specimen changes with the changing of widths for the notch, as shown in Table 7. The predicted values of specimens denoted by “1.5UC” and “2.5UC” are slightly higher than the other three models, which may attribute to the integrity of axial tows in the gauge region. On the other hand, axial fibers among gauge regions of the other three specimens are all incomplete resulting in earlier failure of the specimens than that of specimens with complete fiber bundles.

**Figure 11:**
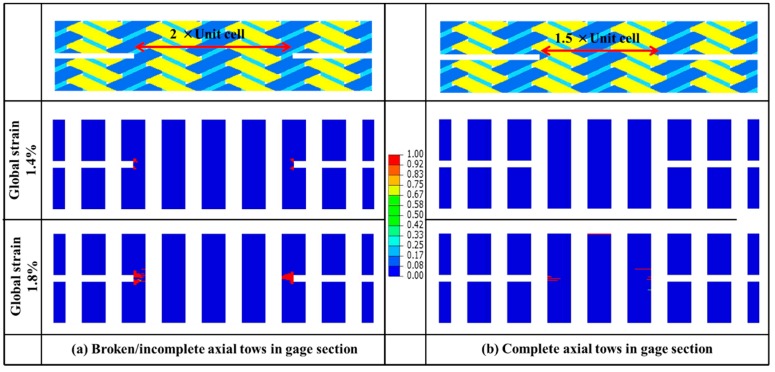
Comparison of predicted damage in in-complete (**a**) and complete (**b**) axial fiber bundles in gauge region.

The fiber damage behavior of two different specimens (“1.5UC” and “2UC”) is shown in Figure 11. As we can see, fiber damage starts in the specimen “2UC” at the global strain level of 1.4%. The damage will spread quickly across the axial fiber bundles of half-width and propagate into the central area. By contrast, fiber damage is not observed in the “1.5UC” specimens at the global strain level of 1.4%. Fiber damage at a global strain level of 1.8% was found to be less significant compared to that in the specimen “2UC”. The results further confirm the necessity of including complete axial fiber bundles in the gauge section when preparing the notched specimens, which could avoid possible variation from cutting and provides more stable and reliable measured properties of the material.

The appropriate design of the specimen is the prerequisite for determining accurately the mechanical properties of braided composites, which is a big challenge due to the lack of advanced test standards. It is an onerous and costly process to optimize the specimen through an experiment only, and the presented numerical method is an alternative and efficient tool for specimen design.

Overall, the fabrics architecture across the gauge section has little influence on the effective strength of the notched sample. However, the simulation capability of the mesomechanical model in predicting the impacts of mesoscale geometric features on the global effective properties of the samples is valuable and advantageous.

## 6. Conclusions

The progressive damage behavior of a notched single-layer triaxially-braided composite under axial tension is analyzed using a three-dimensional mesoscale FE model with anisotropic damage model and an interlaminar tow-to-tow cohesive zone. The proposed model is correlated and validated against full field strain distributions and strength value acquired in the open literature. This mesoscale model is successfully applied to predict the damage propagation of each constituent, including an axial fiber bundle, bias fiber bundle, and interface.

The nonlinearity of global effective stress–strain curve of this notched specimen under axial tension is caused by matrix damage among bias fiber bundles, and the final unloading is identified followed by the fiber tensile damage of axial fiber bundle. The numerical parametric studies identify the sensitivity of stress–strain response to damage parameters. Through geometric characteristic analysis, the different subcell arrangements in the gauge region and dimensions of the notched region are further investigated. The integrity of axial fiber bundles in the test region is considered as the key factor which affects obviously the effective strength of notched specimen. The geometric layout of the subcells in the gauge region shows the negligible impact on the effective strength.

The develop mesoscale FE model could be extremely useful in understanding the failure behavior of this braided composite material. For further studies, this mesoscale model could be used to investigate notched specimens under different loading conditions, like transverse tension. The results of this work demonstrate the feasibility of using a mesoscale FE model as a virtual testing tool framework for braided composites.

## Figures and Tables

**Table 1:** Properties of composite components.

Property	Fiber	Matrix
Material type	T700 s	E862 epoxy
Density (g/cm^3^)	1.8	1.2
Axial modulus (MPa)	230,000	2700
Transverse modulus (MPa)	15,000	2700
Shear modulus (MPa)	27,000	1000
Tensile strength (MPa)	4900	61
Poisson’s ratio	0.2	0.363

**Table 2:** Fracture energies of the fiber bundle.

*G_ft_* (mJ/mm^2^)	*G_fc_* (mJ/mm^2^)	*G_mt_* (mJ/mm^2^)	*G_mc_* (mJ/mm^2^)
12.5	12.5	1	1

**Table 3:** Strengths and fracture toughness of cohesive elements.

*t_n_*^0^ (MPa)	*t_s_*^0^ (MPa)	*t_t_*^0^ (MPa)	*G_n_*^0^ (mJ/mm^2^)	*G_n_*^0^ (mJ/mm^2^)	*G_n_*^0^ (mJ/mm^2^)
122	136	136	0.268	1.45	1.45

**Table 4:** Mechanical properties of axial and bias fiber tows.

	Axial Fiber Tows	Bias Fiber Tows
Fiber volume fraction *V_f_*	86%	69%
*E*_11_ (GPa)	198.18	159.54
*E*_22_ = *E*_33_ (GPa)	11.22	8.30
*G*_12_ = *G*_13_ (GPa)	8.58	4.48
*G*_23_ (GPa)	3.71	2.71
*v*_12_ = *v*_13_	0.29	0.30
*v*_23_	0.51	0.53
*S*_1*t*_ (MPa)	4222	3398.8
*S*_1*c*_ (MPa)	1478.48	1386.21
*S*_2*t*_ = *S*_3*t*_ (MPa)	49.87	49.70
*S*_2*c*_ = *S*_3*c*_ (MPa)	122.80	124.64
*S*_12_ = *S*_13_ = *S*_23_ (MPa)	80.60	78.53

**Table 5:** Comparison of simulation predicted and experimental measured the effective tensile strength of the triaxially-braided composite.

Type of Specimen	Ultimate Strength	
	Simulation (MPa)	Experiment (MPa)
Straight-side coupon	800	814 ± 30 *
Double-notched	751	765 *

* Refer to Kohlman et al. [14].

**Table 6:** The effective strength of notched specimen with different architecture across the gauge section.

Specimen	A-D	B-A	A/2-A/2	B/2-B/2
Strength	764 MPa	772 MPa	751 MPa	768 MPa

**Table 7:** The predicted strength of notched specimens with different geometrical characteristics.

Notch Size	Predicted Strength (MPa)	Section Size	Predicted Strength (MPa)
0.5 × Base	762	1 UC	756
Base	751	1.5 UC *	780 *
2 × Base	755	2 UC	751
		2.5 UC *	772 *
		3 UC	760

* With complete axial tows between notches.
